# Association Between Ceftriaxone Use and Biliary Infections in Patients With Pneumonia: A Nationwide Retrospective Cohort Study

**DOI:** 10.1002/pds.70162

**Published:** 2025-05-21

**Authors:** Jumpei Taniguchi, Shotaro Aso, Hiroki Matsui, Kiyohide Fushimi, Hideo Yasunaga

**Affiliations:** ^1^ Department of Clinical Epidemiology and Health Economics, School of Public Health The University of Tokyo Tokyo Japan; ^2^ Department of Health Services Research, Graduate School of Medicine The University of Tokyo Tokyo Japan; ^3^ Department of Health Policy and Informatics, Graduate School of Medical and Dental Sciences Institute of Science Tokyo Tokyo Japan

**Keywords:** biliary infection, ceftriaxone, cholangitis, cholecystitis, pseudolithiasis

## Abstract

**Background:**

Ceftriaxone is believed to increase the risk of biliary infections due to pseudolithiasis caused by ceftriaxone‐calcium precipitation, but this risk is not well understood. This study aimed to investigate whether ceftriaxone use is associated with an increased risk of biliary infections in pneumonia patients using a national inpatient database.

**Methods:**

We analyzed the Diagnosis Procedure Combination database in Japan, identifying pneumonia patients between July 2010 and March 2022. Patients were grouped by treatment: ceftriaxone versus ampicillin‐sulbactam or cefotaxime. Propensity score overlap‐weighting was used to adjust for confounding factors. The primary outcome was a composite measure, including cholecystitis or cholangitis during hospitalization, and any percutaneous, endoscopic, or surgical interventions on the biliary tract. Secondary outcomes included individual components of the primary outcome.

**Results:**

Of the 1 503 885 eligible patients, 558 725 received ceftriaxone, while 945 160 were treated with either ampicillin‐sulbactam or cefotaxime. The mean dose of ceftriaxone was 1.7 g/day (standard deviation, 0.7 g/day), with a mean administration duration of 7.1 days (standard deviation, 3.8 days). Propensity score overlap‐weighting analysis revealed that ceftriaxone treatment was associated with an increased incidence of the composite outcome (0.22% vs. 0.18%; risk difference, 0.05%; 95% confidence interval, 0.03%–0.07%; *p* < 0.001), as well as the secondary outcomes, including cholecystitis or cholangitis during hospitalization and percutaneous or endoscopic drainage of the gallbladder or biliary tract.

**Conclusion:**

Ceftriaxone use was associated with a slight increase in the risk of biliary infections.

**Summary**

- Ceftriaxone use was associated with a slight but statistically significant increase in the incidence of biliary infections, including cholecystitis and cholangitis, compared with ampicillin‐sulbactam and cefotaxime.
- These results remained consistent across sensitivity analyses using other comparisons.
- Clinicians should be aware of the potential risk.

**Abbreviation**
ICD‐10: International Classification of Diseases 10th revision

## 1 Introduction

Ceftriaxone is a third‐generation cephalosporin with a broad antimicrobial spectrum [1]. It is commonly used for treating a variety of bacterial infections, including pneumonia [2]. Ceftriaxone is excreted in bile, where it accumulates at concentrations 20–150 times higher than that in serum [3]. It binds to calcium ions in bile, inducing reversible precipitation and crystallization [4].

These ceftriaxone‐calcium precipitates had been well studied in pediatric patients [5, 6, 7]. However, recent studies have suggested that pseudolithiasis due to ceftriaxone‐calcium precipitation could also occur in approximately 10%–20% of adult patients [4, 8, 9, 10]. Pseudolithiasis can occasionally lead to biliary infections, such as cholecystitis or cholangitis, which pose a significant clinical concern; however, to what extent ceftriaxone could contribute to the risk of such infections remains unclear [11, 12, 13, 14, 15].

Therefore, this study aims to investigate whether the use of ceftriaxone is associated with an increased risk of cholecystitis and cholangitis in adult patients with pneumonia, using a nationwide inpatient database.

## 2 Methods

### 2.1 Study Design and Setting

This retrospective cohort study utilized the Japanese Diagnosis Procedure Combination inpatient database, which includes comprehensive data from over 1200 hospitals across Japan.

### 2.2 Patient Data

We identified patients diagnosed with pneumonia (International Classification of Diseases, 10th Revision [ICD‐10] codes: J10.x–J18.x, J69.x) between July 2010 and March 2022 from the database. If a patient was hospitalized more than once for pneumonia during the study period, only the first hospitalization was included. We included patients who received ceftriaxone, ampicillin‐sulbactam, or cefotaxime within 2 days of admission. We excluded patients who were treated with ceftriaxone, and ampicillin‐sulbactam or cefotaxime, at the same time; patients with disease of gallbladder (ICD‐10 codes: K56.3, K80.x, K81.x, K82.x, K85.1); patients with diseases of biliary tract (ICD‐10 codes: K74.3, K83.x, K91.8); patients with malignant neoplasm of gallbladder or biliary tract (ICD‐10 codes: C22.1, C23.x, C24.x, D13.4, D13.5, D37.6); patients with medical history of cholecystectomy (ICD‐10 codes: K91.5, Z90.4); patients treated with ursodiol within 2 days of admission; patients aged < 15 years; and pregnant patients.

### 2.3 Data Source

We utilized the Japanese Diagnosis Procedure Combination inpatient database containing comprehensive data on patient demographics (age, sex, height, and weight), primary diagnosis, comorbidities at admission, complications arising during hospitalization (coded according to the ICD‐10), dates of admission and discharge, prescribed medications, procedures, surgeries, and hospital identification numbers [16]. This database holds detailed administrative and discharge records from over 1200 hospitals across Japan [17].

### 2.4 Exposure

Patients were divided into two groups: those who received ceftriaxone (the ceftriaxone group) and those who received ampicillin‐sulbactam or cefotaxime (the control group) within 2 days of admission. The antimicrobial spectra of ampicillin‐sulbactam and cefotaxime are similar to that of ceftriaxone; both are widely used for the treatment of community‐acquired pneumonia. Ceftriaxone is known to be excreted into the gallbladder, potentially leading to the formation of ceftriaxone‐calcium precipitates, while ampicillin‐sulbactam and cefotaxime are primarily excreted by the kidneys, without reports of inducing calcium precipitate formation. Therefore, ampicillin‐sulbactam and cefotaxime were selected as comparators.

### 2.5 Covariates and Outcomes

Covariates were classified into three categories: baseline patient characteristics, treatments received within 2 days of admission, and hospital characteristics. Baseline patient characteristics included factors such as age, sex, body mass index at admission, smoking status (nonsmoker, current or former smoker), Glasgow Coma Scale score at admission, Barthel Index [18], whether the patient was admitted to an intensive care unit or high care unit, and comorbidities (including lung diseases [chronic obstructive pulmonary disease, interstitial pneumonia, bronchiectasis or non‐tuberculosis mycobacterium of lungs, fungal lung disease, lung tumors, chronic respiratory failure], cardiovascular disease, chronic kidney disease, liver disease, diabetes mellitus, dyslipidemia, non‐hematological malignancies, hematological malignancies, and dementia) (Table S1). Treatments received within the 2 days of admission included oxygen therapy, mechanical ventilation, the use of vasopressors, renal replacement therapy, oral feeding, nasogastric feeding, total parenteral nutrition, medications associated with gallstone formation (e.g., fibrates, somatostatin analogs, and hormone replacement), antibiotics (e.g., macrolides, fluoroquinolones, doxycycline, and anti‐methicillin‐resistant *Staphylococcus aureus* ), and steroids. Hospital characteristics included whether the patient was admitted to a teaching hospital.

Body mass index was categorized into the following groups: < 18.5, 18.5–24.9, 25.0–29.9, and ≥ 30.0 kg/m^2^. The Japan Coma Scale score was converted to the Glasgow Coma Scale score based on a prior study [19], as the two scales have been shown to be well‐correlated [20]. The Barthel Index was categorized into four groups: 0, 5–50, 55–95, and 100. Steroid use was defined as the administration of at least 200 mg/day of hydrocortisone or an equivalent dose of another corticosteroid.

The primary outcome was defined as a composite of the following: cholecystitis or cholangitis (ICD‐10 codes: K80.0, K80.1, K80.3, K80.4, K81.x, K83.0), percutaneous or endoscopic drainage of the gallbladder or biliary tract, and surgical interventions on the gallbladder or biliary tract during hospitalization. Secondary outcomes were defined as each individual component of the primary outcome.

### 2.6 Statistical Analyses

We applied a propensity‐score overlap‐weighting method to compare the outcomes between the two groups [21, 22]. Propensity scores were derived from a logistic regression model, with the dependent variable being ceftriaxone use and all covariates included as independent variables. Overlap weighting involves assigning weights to patients based on their likelihood of receiving the opposing treatment [22]. In this study, patients treated with ceftriaxone were weighted by the probability of not receiving ceftriaxone (1 − propensity score), while those who did not receive ceftriaxone were weighted by their probability of receiving ceftriaxone (propensity score). This overlap‐weighting approach helps balance covariates between groups, mimicking the conditions of a randomized trial without excluding any patients from the analysis. Standardized differences for each covariate were computed, with absolute standardized differences less than 10% indicating adequate balance [23, 24].

For statistical analysis, continuous variables were evaluated using *t*‐tests and are expressed as means with standard deviations. Categorical variables were analyzed using the chi‐square test and are presented as frequencies and percentages. A two‐sided *p*‐value of < 0.05 was considered statistically significant. All analyses were conducted using STATA/SE version 18.0 (StataCorp, College Station, TX, USA).

### 2.7 Sensitivity Analysis

We conducted several sensitivity analyses. First, we restricted the analysis to patients receiving a high dose of ceftriaxone (≥ 2 g/day) in the ceftriaxone group [25]. Second, we compared the outcomes between ceftriaxone and piperacillin‐tazobactam as an alternative control. Third, we restricted the control group to patients who received cefotaxime, which has an antibacterial spectrum similar to ceftriaxone but is primarily excreted renally. Fourth, we performed an additional analysis that included patients who were readmitted for biliary infections within 30 days after discharge to account for the possibility that outcomes might have occurred after discharge.

### 2.8 Ethics

This study received ethical approval from the Institutional Review Board of the University of Tokyo (3501‐(5), May 19th, 2021). Written informed consent was waived due to the retrospective design and use of anonymized data.

## 3 Results

**FIGURE 1:**
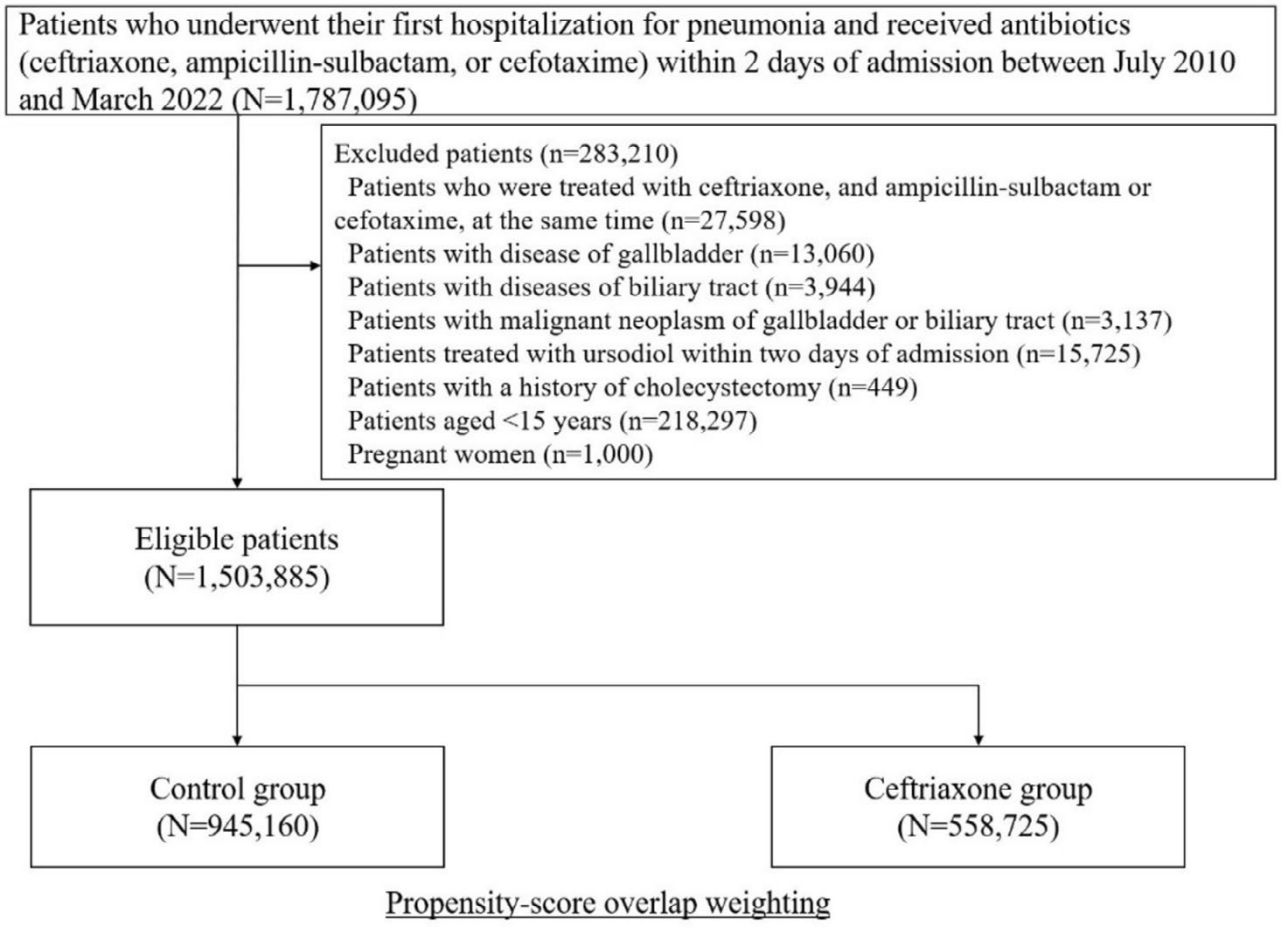
Study design. This flowchart illustrates the inclusion and exclusion criteria for the study. A total of 1 787 095 patients who underwent their first hospitalization for pneumonia and received antibiotics (ceftriaxone, ampicillin‐sulbactam, or cefotaxime) within 2 days of admission between July 2010 and March 2022 were screened. After excluding 283 210 patients based on criteria such as concurrent antibiotic treatment, gallbladder or biliary tract disease, malignancies, use of ursodiol, previous cholecystectomy, age < 15 years, and pregnancy, 1 503 885 patients were eligible for the study. Eligible patients were divided into two groups: Ceftriaxone group (*N* = 558 725) and control group (*N* = 945 160).

We identified 1 787 095 patients who were hospitalized for pneumonia and received antibiotics, including ampicillin‐sulbactam, cefotaxime, or ceftriaxone, between July 2010 and March 2022 (Figure 1). Of these, 1 503 885 patients were eligible for inclusion in the analysis, with 558 725 and 945 160 patients in the ceftriaxone and control groups, respectively. In the ceftriaxone group, the mean initial dose of ceftriaxone was 1.7 g/day (standard deviation, 0.7 g/day); the mean duration of administration was 7.1 days (standard deviation, 3.8 days). In the control group, the mean duration of administration was 7.7 days (standard deviation, 4.1 days). The mean length of hospital stay was 20 (standard deviation, 30 days) and 23 (standard deviation, 31 days) days in the ceftriaxone and control groups, respectively.

Table 1 shows the baseline characteristics of the study groups before and after propensity‐score overlap weighting. Compared with the control group, patients in the ceftriaxone group were younger, had a higher BMI, had less altered consciousness, had better Barthel Index scores, were more likely to have chronic kidney failure, and were more likely to receive oral feeding and macrolide treatment within 2 days of admission. In the unweighted population, there were no significant differences in the proportions of the composite outcome between the ceftriaxone and control groups (0.21% [1181/558 725] vs. 0.20% [1903/945 160]; risk difference [RD], 0.01%; 95% confidence interval [CI], −0.00% to 0.03%; *p* = 0.189).

**TABLE 1:** Patient characteristics.

Variables	Unweighted population			Propensity‐score overlap weighted population		
	Control (*n* = 945 160)	Ceftriaxone (*n* = 558 725)	Standardized difference	Control	Ceftriaxone	Standardized difference
Age, years, mean (SD)	79.9 (13.6)	77.4 (15.4)	−17.6	77.6 (15.2)	77.6 (15.3)	0.0
Male, *n* (%)	533 288 (56.4)	315 800 (56.5)	0.2	(55.7)	(55.7)	0.0
BMI, kg/m^2^, *n* (%)						
< 18.50	298 426 (31.6)	146 992 (26.3)	−11.6	(32.3)	(32.3)	0.0
18.50–24.99	416 181 (44.0)	269 418 (48.2)	8.4	(54.1)	(54.1)	0.0
25.00–29.99	75 948 (8.0)	59 912 (10.7)	9.2	(11.1)	(11.1)	0.0
≥ 30.00	14 972 (1.6)	13 955 (2.5)	6.5	(2.4)	(2.4)	0.0
Missing data	139 633 (14.8)	68 448 (12.3)	−7.4			
Smoking history, *n* (%)						
Patient who does not smoke	585 929 (62.0)	334 100 (59.8)	−4.5	(68.5)	(68.5)	0.0
Patient who currently smokes/patient who previously smoked	230 682 (24.4)	155 989 (27.9)	8.0	(31.5)	(31.5)	0.0
Missing data	128 549 (13.6)	68 636 (12.3)	−3.9			
GCS on admission, mean (SD)	14.1 (2.1)	14.4 (1.7)	16.6	14.4 (1.7)	14.4 (1.7)	0.0
Barthel index on admission, *n* (%)						
0	345 195 (36.5)	142 788 (25.6)	−23.9	(31.5)	(31.5)	0.0
5–50	186 228 (19.7)	108 475 (19.4)	−0.7	(22.8)	(22.8)	0.0
55–95	99 369 (10.5)	77 586 (13.9)	10.3	(15.6)	(15.6)	0.0
100	180 351 (19.1)	153 925 (27.5)	20.1	(30.1)	(30.1)	0.0
Missing data	134 017 (14.2)	75 951 (13.6)	−1.7			
ICU or HCU admission, *n* (%)	36 116 (3.8)	17 968 (3.2)	−3.3	(3.3)	(3.3)	0.0
Lung disease, *n* (%)						
Chronic obstructive pulmonary disease	67 272 (7.1)	48 235 (8.6)	5.6	(8.2)	(8.2)	0.0
Interstitial Pneumonia	21 205 (2.2)	16 693 (3.0)	4.7	(2.8)	(2.8)	0.0
Bronchiectasis & NTM of the lungs	10 065 (1.1)	7107 (1.3)	1.9	(1.3)	(1.3)	0.0
Fungal lung disease	2456 (0.3)	1585 (0.3)	0.5	(0.3)	(0.3)	0.0
Lung tumors	31 806 (3.4)	17 294 (3.1)	−1.4	(3.1)	(3.1)	0.0
Chronic respiratory failure	18 344 (1.9)	11 608 (2.1)	1.0	(2.0)	(2.0)	0.0
Cardiovascular disease, *n* (%)	161 652 (17.1)	106 155 (19.0)	4.9	(18.2)	(18.2)	0.0
Chronic kidney failure, *n* (%)	38 100 (4.0)	42 011 (7.5)	14.9	(5.7)	(5.7)	0.0
Liver disease, *n* (%)	24 880 (2.6)	15 267 (2.7)	0.6	(2.9)	(2.9)	0.0
Diabetes mellitus, *n* (%)	151 649 (16.0)	102 551 (18.4)	6.1	(17.9)	(17.9)	0.0
Dyslipidemia, *n* (%)	65 057 (6.9)	45 718 (8.2)	4.9	(8.2)	(8.2)	0.0
Non‐hematological malignancy, *n* (%)	74 560 (7.9)	38 960 (7.0)	−3.5	(7.7)	(7.7)	0.0
Hematological malignancy, *n* (%)	6422 (0.7)	6270 (1.1)	4.7	(1.0)	(1.0)	0.0
Dementia, *n* (%)	161 789 (17.1)	71 595 (12.8)	−12.1	(13.9)	(13.9)	0.0
Treatment within 2 days of admission day, *n* (%)						
Oxygenation	556 927 (58.9)	294 768 (52.8)	−12.4	(52.4)	(52.4)	0.0
Mechanical ventilation	29 699 (3.1)	15 492 (2.8)	−2.2	(2.6)	(2.6)	0.0
Vasopressors	22 531 (2.4)	13 236 (2.4)	−0.1	(2.1)	(2.1)	0.0
Renal replacement therapy	7341 (0.8)	9502 (1.7)	8.4	(1.2)	(1.2)	0.0
Oral feeding	557 843 (59.0)	418 858 (75.0)	34.4	(72.3)	(72.3)	0.0
Tube feeding	17 320 (1.8)	6689 (1.2)	−5.2	(1.4)	(1.4)	0.0
Total parenteral nutrition	7540 (0.8)	2516 (0.5)	−4.4	(0.5)	(0.5)	0.0
Fibrates	2131 (0.2)	1923 (0.3)	2.2	(0.3)	(0.3)	0.0
Somatostatin analogs	38 (0.0)	20 (0.0)	0.7	(0.0)	(0.0)	0.0
Hormone replacement	243 (0.0)	218 (0.0)	0.7	(0.0)	(0.0)	0.0
Macrolide	58 964 (6.2)	77 398 (13.9)	25.5	(10.4)	(10.4)	0.0
Fluoroquinolone	24 336 (2.6)	23 893 (4.3)	9.4	(3.7)	(3.7)	0.0
Doxycycline	13 313 (1.4)	14 578 (2.6)	8.6	(2.1)	(2.1)	0.0
Anti‐MRSA	2334 (0.2)	2663 (0.5)	3.8	(0.3)	(0.3)	0.0
Steroids	45 232 (4.8)	35 115 (6.3)	6.6	(5.5)	(5.5)	0.0
Teaching hospital admission, *n* (%)	758 495 (80.3)	443 436 (79.4)	−2.2	(77.8)	(77.8)	0.0

*Note:* The total number of etiologies does not add up to 100% as more than one cause can be assigned to a single patient.

Abbreviations: BMI, body mass index; GCS, Glasgow Coma Scale; HCU, high care unit; ICU, intensive care unit; MRSA, methicillin resistance in *Staphylococcus aureus* ; NTM, non‐tuberculosis mycobacterium; SD, standard deviation.

After applying propensity‐score overlap weighting, all baseline characteristics were well‐balanced (Table 1). In the weighted population, the proportion of the composite outcome was higher in the ceftriaxone group than in the control group (0.22% vs. 0.18%; RD, 0.05%; 95% CI, 0.03%–0.07%; *p* < 0.001) (Table 2). The risk ratio was 1.27; the number needed to harm was 2079. The proportions of the following secondary outcomes were also higher in the ceftriaxone group: complications of cholecystitis or cholangitis during hospitalization and percutaneous or endoscopic drainage of the gallbladder or biliary tract (Table 2).

**TABLE 2:** Comparison of outcomes between groups.

	Control	Ceftriaxone	Risk difference	95% confidence interval	*p*
Primary outcome, %					
Crude	0.20	0.21	0.01	−0.00 to 0.03	0.189
Propensity score overlap weighting	0.18	0.22	0.05	0.03 to 0.07	< 0.001
Secondary outcome, %					
Complication of cholecystitis or cholangitis during hospitalization					
Crude	0.17	0.17	0.01	−0.00 to 0.02	0.205
Propensity score overlap weighting	0.14	0.19	0.04	0.03 to 0.06	< 0.001
Percutaneous or endoscopic drainage on the gallbladder or biliary tract					
Crude	0.06	0.06	0.01	−0.00 to 0.01	0.174
Propensity score overlap weighting	0.05	0.07	0.01	0.00 to 0.02	0.005
Surgical interventions on the gallbladder or biliary tract					
Crude	0.01	0.01	0.00	−0.00 to 0.00	0.994
Propensity score overlap weighting	0.01	0.01	0.00	−0.00 to 0.00	0.899

*Note:* Primary outcome was defined as a composite outcome of the complications of cholecystitis or cholangitis during hospitalization, percutaneous or endoscopic drainage on the gallbladder or biliary tract, and surgical interventions on the gallbladder or biliary tract.

Table 3 shows the results of the sensitivity analyses. Patients who received high‐dose ceftriaxone (≥ 2 g/day) had a higher proportion of the primary outcome compared to those who received ampicillin‐sulbactam or cefotaxime (0.22% vs. 0.18%; RD, 0.04%; 95% CI, 0.02%–0.06%; *p* < 0.001). When comparing ceftriaxone to piperacillin‐tazobactam, the primary outcome was consistent with the main analysis (0.25% vs. 0.19%; RD, 0.05%; 95% CI, 0.02%–0.08%; *p* < 0.001). In the comparison between ceftriaxone and cefotaxime, there was no significant difference in the primary outcome (0.18% vs. 0.15%; RD, 0.03%; 95% CI, −0.04% to 0.10%; *p* = 0.416). However, percutaneous or endoscopic drainage of the gallbladder or biliary tract was significantly more frequent in the ceftriaxone group (0.05% vs. 0.01%; RD, 0.04%; 95% CI, 0.02%–0.07%; *p* < 0.001). The results of the sensitivity analysis including 30‐day readmissions for biliary infections were consistent with those of the main analysis (Table S2).

**TABLE 3:** Sensitivity analyses of outcomes between propensity score weighted groups.

	Risk difference	95% confidence interval	*p*
Ceftriaxone ≥ 2 g/day (*n* = 361 737) vs. ampicillin‐sulbactam or cefotaxime (*n* = 945 160)			
Primary outcome, %	0.04	0.02 to 0.06	< 0.001
Secondary outcomes, %			
Complication of cholecystitis or cholangitis during hospitalization	0.04	0.02 to 0.06	< 0.001
Percutaneous or endoscopic drainage on the gallbladder or biliary tract	0.01	0.00 to 0.03	0.028
Surgical interventions on the gallbladder or biliary tract	0.00	−0.01 to 0.00	0.682
Ceftriaxone (*n* = 573 867) vs. piperacillin‐tazobactam (*n* = 318 510)			
Primary outcome, %	0.05	0.02 to 0.08	< 0.001
Secondary outcomes, %			
Complication of cholecystitis or cholangitis during hospitalization	0.06	0.03 to 0.08	< 0.001
Percutaneous or endoscopic drainage on the gallbladder or biliary tract	0.01	−0.01 to 0.02	0.360
Surgical interventions on the gallbladder or biliary tract	0.01	−0.00 to 0.01	0.059
Ceftriaxone (*n* = 558 725) vs. cefotaxime (*n* = 16 303)			
Primary outcome, %	0.03	−0.04 to 0.10	0.416
Secondary outcomes, %			
Complication of cholecystitis or cholangitis during hospitalization	0.01	−0.06 to 0.08	0.762
Percutaneous or endoscopic drainage on the gallbladder or biliary tract	0.04	0.02 to 0.07	< 0.001
Surgical interventions on the gallbladder or biliary tract	0.00	−0.02 to 0.02	0.952

*Note:* Primary outcome was defined as a composite outcome of the complications of cholecystitis or cholangitis during hospitalization, percutaneous or endoscopic drainage on the gallbladder or biliary tract, and surgical interventions on the gallbladder or biliary tract.

## 4 Discussion

In this retrospective study, we evaluated the association between ceftriaxone use and biliary infection in patients with pneumonia. Our findings revealed that the use of ceftriaxone was associated with a slight increase in the risk of biliary infection in these patients.

A major strength of the present study is the use of a large‐scale nationwide inpatient database. Previous studies investigating the association between ceftriaxone and biliary infections have been primarily limited to case reports. To date, no comprehensive analyses have been conducted to evaluate the clinical significance of ceftriaxone‐calcium precipitation [11, 12, 13]. In contrast, our study aimed to address this critical gap by assessing the association between ceftriaxone use and biliary infections among patients with pneumonia using a large nationwide database. To the best of our knowledge, this study is the first large‐scale comprehensive study to investigate the association and provides valuable epidemiological evidence on the potential risk of biliary complications with ceftriaxone use.

The exact pathogenic mechanism of pseudolithiasis remains unclear. It is hypothesized that ceftriaxone‐calcium precipitation in the bile leads to the formation of pseudolithiasis [4]. Other studies suggest that ceftriaxone may reduce gallbladder contractility or that genetic polymorphisms in the uridine 5‐diphosphate‐glucuronosyltransferase 1A1 gene may contribute to stone formation [26, 27]. A previous double‐blind, placebo‐controlled study found that 21.4% of adults developed pseudolithiasis after receiving intravenous ceftriaxone at 2 g/day for 14 days [4]. Additionally, a large‐scale retrospective study reported that pseudolithiasis developed in 17% (89/523) of adult patients who received a total ceftriaxone dose of 9.1 g (standard deviation, 12.4 g) over a median treatment duration of 3 days (interquartile range, 1–66 days) [25]. Since pseudolithiasis typically resolves spontaneously within 9–26 days after discontinuation of ceftriaxone, it remains uncertain whether ceftriaxone‐induced pseudolithiasis significantly increases the risk of biliary diseases in patients receiving standard treatment for pneumonia [4].

In our study, the mean initial dose of ceftriaxone was 1.7 g/day (standard deviation, 0.7 g), and the mean duration of administration was 7.1 days (standard deviation, 3.8 days). Although ceftriaxone use was associated with a slight increase in the risk of biliary infections, this risk should be weighed against the benefits of ceftriaxone in treating pneumonia, including its broad antimicrobial spectrum, convenience of once‐daily dosing, and availability. Considering the relatively small increase in risk, it may not be necessary to focus heavily on biliary complications when using ceftriaxone in standard clinical practice for pneumonia management [28].

This study has some limitations. First, this was a retrospective analysis based on a nationwide database. Due to the nature of the database, which is primarily used for reimbursement purposes, detailed clinical information, such as laboratory test results and imaging findings, was not available. Although complications and surgical procedures are generally expected to be recorded with high specificity due to using reimbursement claims, the accuracy of diagnostic coding for specific conditions such as cholecystitis and cholangitis may not be fully confirmed. Furthermore, we could not rule out the risk of misclassification bias regarding whether the included procedures, such as drainage and surgical interventions in the gallbladder or biliary tract, could have been performed for other indications. Additionally, although we adjusted for several measured confounders, the potential impact of unmeasured confounders could not be excluded. These limitations may have affected the internal validity of our findings. Second, we excluded patients with pre‐existing gallbladder or biliary tract disease. As a result, the impact of ceftriaxone use on the risk of biliary infections in patients with such coexisting conditions remains unclear. Third, this study was based on an inpatient database, and outcomes that occurred after discharge could not be fully assessed. Ceftriaxone‐induced pseudolithiasis is generally expected to resolve spontaneously after ceftriaxone discontinuation. We also performed a sensitivity analysis that included patients who were readmitted for biliary infections within 30 days after discharge and confirmed the robustness of our findings. However, it remains possible that not all outcomes might have been fully assessed, which might have led to an underestimation of the true incidence of biliary complications.

## 5 Conclusion

This study found that ceftriaxone use in patients with pneumonia was associated with a slight increase in the risk of biliary infections. Although this risk is statistically significant, it is relatively small in magnitude. Clinicians should be aware of this risk, but given the broad benefits of ceftriaxone in treating pneumonia, it may not warrant significant changes in clinical practice for most patients.

### 5.1 Plain Language Summary

Ceftriaxone is a common antibiotic used to treat infections including pneumonia. However, it can sometimes cause a condition called pseudolithiasis, where calcium builds up and forms stone‐like deposits in the gallbladder or bile ducts. This may increase the risk of biliary infections, such as cholecystitis (inflammation of the gallbladder) and cholangitis (inflammation of the bile ducts). In this study, we analyzed a large national database of hospitalized pneumonia patients in Japan from 2010 to 2022. We compared patients who received ceftriaxone with those treated with other antibiotics like ampicillin‐sulbactam or cefotaxime. Our analysis found that patients treated with ceftriaxone had a slightly higher risk of developing biliary infections or needing drainage procedures during their hospital stay (0.22% vs. 0.18%). Although this difference was statistically significant, the overall risk was very low. This information can help healthcare providers make more informed decisions when prescribing antibiotics, especially for patients who may be more susceptible to biliary problems.

## Author Contributions

J.T. conceived, designed, analyzed, and coordinated this study. J.T. and S.A. wrote the first draft of the manuscript. H.Y. contributed to the data interpretation and assisted with the manuscript preparation. S.A., H.M., K.F., and H.Y. critically reviewed the manuscript. H.Y. critically appraised the manuscript. All the authors have read and approved the final version of the manuscript.

## Conflicts of Interest

The authors declare no conflicts of interest.

## Supporting information


**Table S1.** ICD‐10 codes used to define each comorbidity.
**Table S2.** Sensitivity analysis including patients who were readmitted for biliary infections within 30 days after discharge.

## Data Availability

The datasets used in this study are not available.
