# Supplementary material for: Association Between Ceftriaxone Use and Biliary Infections in Patients With Pneumonia: A Nationwide Retrospective Cohort Study
Source: Pharmacoepidemiol Drug Saf. 2025 May 21;34(6):e70162. doi: 10.1002/pds.70162 (PMC12094858; doi:10.1002/pds.70162)
Supplement: Supplementary file 1 — Table S1. ICD‐10 codes used to define each comorbidity. Table S2. Sensitivity analysis including patients who were readmitted for biliary infections within 30 days after discharge. [file PDS-34-e70162-s001.docx]

**Supplementary Table 1**. **ICD-10 codes used to define each comorbidity**

| Chronic obstructive pulmonary disease | J43.x, J44.x |
| --- | --- |
| Interstitial Pneumonia | J84.x, J99.x |
| Bronchiectasis & NTM of the lungs | A31.0, J47.x |
| Fungal lung disease | B37.1, B38.0, B38.1, B38.2, B39.0, B39.1, B39.2, B40.0, B40.1, B40.2, B41.0, B42.0, B44.0, B44.1, B45.0, B46.0 |
| Lung tumors | C34.x |
| Chronic respiratory failure | J96.1 |
| Cardiovascular disease | I20.x–25.x, I42.x, I50.x |
| Chronic kidney failure | I12.0, N18.x–19.x |
| Liver disease | B16.x–19.x, K70.x–77.x |
| Diabetes mellites | E10.x–E14.x |
| Dyslipidemia | E78.x |
| Non-hematologic malignancy | C00.x–33.x, C35.x–80.x, C97.x, D00.x–09.x |
| Hematologic malignancy | C81.x–96.x, T86.0, T86.8, T86.9, Y83.8, Z94.8, Z94.9 |
| Dementia | F00.x–F03.x, G30.x |

**Supplementary Table 2.** **Sensitivity analysis including patients who were readmitted for biliary infections within 30 days after discharge**

|  | Control | Ceftriaxone | Risk difference | 95% confidence interval | P |
| --- | --- | --- | --- | --- | --- |
| Primary outcome, % | 0.22 | 0.29 | 0.07 | 0.05 to 0.09 | <0.001 |
